# Timing of perinatal death; causes, circumstances, and regional variations among reviewed deaths in Ethiopia

**DOI:** 10.1371/journal.pone.0285465

**Published:** 2023-05-09

**Authors:** Neamin Tesfay, Rozina Tariku, Alemu Zenebe, Girmay Hailu, Muse Taddese, Fitsum Woldeyohannes

**Affiliations:** 1 Centre of Public Health Emergency Management, Ethiopian Public Health Institutes, Addis Ababa, Ethiopia; 2 Health Financing Program, Clinton Health Access Initiative, Addis Ababa, Ethiopia; Arba Minch University, ETHIOPIA

## Abstract

**Introduction:**

Ethiopia is one of the countries facing a very high burden of perinatal death in the world. Despite taking several measures to reduce the burden of stillbirth, the pace of decline was not that satisfactory. Although limited perinatal mortality studies were conducted at a national level, none of the studies stressed the timing of perinatal death. Thus, this study is aimed at determining the magnitude and risk factors that are associated with the timing of perinatal death in Ethiopia.

**Methods:**

National perinatal death surveillance data were used in the study. A total of 3814 reviewed perinatal deaths were included in the study. Multilevel multinomial analysis was employed to examine factors associated with the timing of perinatal death in Ethiopia. The final model was reported through the adjusted relative risk ratio with its 95% Confidence Interval, and variables with a p-value less than 0.05 were declared statistically significant predictors of the timing of perinatal death. Finally, a multi-group analysis was carried out to observe inter-regional variation among selected predictors.

**Result:**

Among the reviewed perinatal deaths, 62.8% occurred during the neonatal period followed by intrapartum stillbirth, unknown time of stillbirth, and antepartum stillbirth, each contributing 17.5%,14.3%, and 5.4% of perinatal deaths, respectively. Maternal age, place of delivery, maternal health condition, antennal visit, maternal education, cause of death (infection and congenital and chromosomal abnormalities), and delay to decide to seek care were individual-level factors significantly associated with the timing of perinatal death. While delay reaching a health facility, delay to receive optimal care health facility, type of health facility and type region were provincial-level factors correlated with the timing of perinatal death. A statistically significant inter-regional variation was observed due to infection and congenital anomalies in determining the timing of perinatal death

**Conclusion:**

Six out of ten perinatal deaths occurred during the neonatal period, and the timing of perinatal death was determined by neonatal, maternal, and facility factors. As a way forward, a concerted effort is needed to improve the community awareness of institutional delivery and ANC visit. Moreover, strengthening the facility level readiness in availing quality service through all paths of the continuum of care with special attention to the lower-level facilities and selected poor-performing regions is mandatory.

## Introduction

Perinatal mortality rate is one of the pivotal parameters used to assess the overall health status of a defined society [1]. It is a multifactorial and multifaceted etiology and has implications on a continuum of care, extending from pre-conception up to post-partum care [1, 2]. Globally, an estimated 3.8 million perinatal deaths were reported in 2019 [2, 3]. The burden of perinatal death was prominent in low and middle-income countries [4]. A recent study on sub-Saharan African countries indicated that the estimated perinatal mortality rate was 34.7 per 1,000 births [5, 6], which is much higher than the global estimate of 26.7 deaths per 1,000 births [2].

Several global initiatives were designed to improve perinatal outcomes; Every Newborn Action Plan (ENAP) is one of the global strategies designed to address the quality of care at birth by generating data for decision-making and action. In addition, strategies for ending preventable maternal mortality were also put in place to reduce inequality in service access and provision [7]. To consolidate these efforts, a new global target was established under the sustainable development goal (SDG), which has set a goal of reducing stillbirth and neonatal mortality to only 12 deaths per 1000 livebirth by 2030 [8].

Ethiopia is one of the top perinatal death reporting countries in the world [2]. The country has made progress in the reduction of the burden of maternal and perinatal death in the last two decades [9, 10]. According to the Ethiopian Demographic and Health Survey (EDHS), the estimated perinatal death is around 33 deaths per 1000 live birth, with notable regional variation [11].

One of the key recommendations that the World Health Organization(WHO) put in place to reduce the burden of maternal and child mortality is understanding the number, and cause of death by establishing a nationwide surveillance system [12]. Per the recommendation, Ethiopia established Maternal Death Surveillance and Response (MDSR) system in 2013, and later in 2017, Perinatal Death Surveillance and Response (PDSR) was implemented by integrating perinatal death into the existing MDSR system [13]. Later in 2017, the name of the system was changed to the Maternal and Perinatal Death Surveillance and Response (MPDSR) system incorporating both maternal and perinatal components [14].

PDSR system is a continuous-surveillance cycle designed to provide real-time, actionable data on perinatal mortality levels, causes of death, and contributing factors, with a focus on using the findings to plan appropriate and effective preventive actions [15]. PDSR operates at both facility and community levels; however, the system was not fully implemented due to different factors, both at community (low engagement) and health facility (poor attendance, defensive attitude, and blaming shifting among health professionals) level [14, 15].

In addition to establishing the MDPSR system, Ethiopia has taken different measures to tackle the burden of perinatal death, among those the implementation of a mandatory 24 hours stay at a health facility after delivery [16], the scaling up of integrated community case management (iCCM) and community-based newborn care (CBNC) [17, 18] and improving the readiness and access to comprehensive emergency maternal and newborn care are some of the measures taken in this front [19]. Furthermore, the country has made strides at strengthening and expanding advanced neonatal care, neonatal intensive care unit (NICU), and Essential New-born Care (ENBC) services including services for low birth weight and preterm neonates [20, 21]. On top of this, interventions such as introducing micronutrient supplementation as a maternal nutritional program [22], enhancing the quality of antenatal care [23], improving the management of sexually transmitted infections [24], and improving access to services aimed at preventing Mother to Child Transmission of HIV [25] and enhancing awareness on the utilization of insecticide-treated bed net during pregnancy [26] were taken as a specific measure to reduce the burden of stillbirth Ethiopia. Despite all these efforts, the reduction rate of perinatal mortality is not that satisfactory [27].

Understating the timing of perinatal death has paramount importance in planning health programs and setting priorities [28]. The timing of perinatal death has a four-time classification based on labor and delivery. Those are 1) death that has happened before the onset of labour (antepartum stillbirth), 2) death that happened during the onset of labour (intrapartum stillbirth), 3) unknown time of fetal death before birth (unknown stillbirths), and 4) death occurring after delivery (neonatal death) [29]. Globally, an estimated 42·3% of all stillbirths are intrapartum; while 75% of neonatal death occurred during the early neonatal period and almost all of these can be prevented with timely, and quality care during labour and post-delivery period [3, 30].

Perinatal death is one of the key indicators showcasing the status of the continuum of care provided in the country. The fetal outcome during perinatal time is influenced by the individual (fetal and maternal condition) and facility-level factors [31]. Gestational age, congenital anomaly, neonatal infection, birth weight, presentation during delivery, mode of delivery, and multiple pregnancies are the fetal factors that contribute to perinatal death [32–38]; while, maternal age, marital status, maternal parity, history of antennal care (ANC), wealth status, maternal education, pre-existing maternal health condition, and smoking status are the potential maternal factor that contributes to perinatal death [39–43]. Furthermore, travel time to reach a health facility, length of stay in a health facility, lack of competent staff, and capacity of diagnosis are some of the facility-level factors that contribute to perinatal death [44–47].

Despite the high burden of perinatal death in Ethiopia, the topic was not well explored using national mortality surveillance data. Thus, this study is aimed at identifying potential factors related to the timing of perinatal death by incorporating both individual and facility-level factors among nationally reviewed perinatal deaths.

## Methods

### Study setting

Ethiopia has an estimated population of 117,876,000 in 2021, out of which 17, 216,372 are under-five children [48]. Administratively, Ethiopia has ten regions and two city administrations, namely Tigray, Afar, Amhara, Oromia, Somali, Benishangul-Gumuz, Southern Nations Nationalities, and Peoples Region (SNNPR), Sidama, Gambella, Harari, Addis Ababa city administration and Dire Dawa city administration [49]. The country has high infant, under-five, and maternal mortality (47 per 1000LBs), (59 per 1000LBs), and (412 per 100,00 LBs), respectively [11, 50].

### Data source and study participant

The study used data from Ethiopian Public Health Institutes (EPHI), which is collected and compiled from various health facilities across Ethiopia. It utilized an updated programmatical and epidemiological review of perinatal death data obtained from all PDSR implementing regions for four consecutive years (2018–2021). The data was extracted through facility-based abstraction format (FBAF) and verbal autopsy (VA). The source population for the study is all perinate who died and were reviewed by the MPDSR committee during the study period. Accordingly, a total of 3814 reviewed perinatal deaths were included in the study. The PDSR data was hierarchical i.e., perinate was nested in 161 reporting health facilities and 45 provinces of the country.

### Study variables

#### Outcome variable

The dependent variable is the time of perinatal death relating to labour and delivery. The multinomial dependent variable was classified as “Antepartum” (deceased before delivery), “Intrapartum” (deceased during delivery), “Stillbirth of unknown time” (undefined time of death before delivery), and “Neonatal death” (deceased after delivery within 28 days).

#### Explanatory variables

Both individual (neonatal and maternal factor) and facility-level variables were included as a predictor in the model. Sex, gestational age, place of birth, mode of delivery, and assigned cause of death were included as neonatal factors in the model. The medical cause of death was incorporated as individual death after the underlying cause of death was assigned using the International Classification of Diseases-Perinatal Mortality (ICD-PM) [51]. From the maternal factors, variables such as maternal age, maternal parity, educational status, number of ANC (antenatal care) visits, a score of delay one, and maternal health condition were included in the model. Moreover, maternal health conditions were assigned per the guidance of ICD-PM. The score of delay one, which is a delay in deciding to seek care [52], was computed using the row sum of seven variables included under this domain; namely 1) family poverty, 2) bad experience with previous health service, 3) failure to recognize the danger signs of pregnancy, 4) lack of awareness on where to seek care, 5) lack of a person who could take care of other children, 6) reliant on traditional practice, and 7) lack of decision to go to a health facility. All of them were binary variables with ‘Yes’ and ‘No’ responses and after summation of the score, to keep the normality of the data, a square root transformation was conducted [53]. Finally, the transformed variable was treated as continuous variables to make a parsimonious model [54]. At a facility (community) level; variables such as residence, type of region, type of health facility, the score of delay two, and delay three were taken into consideration. The type of region was classified into three categories (city, agrarian, and pastoralist) based on the cultural and socio-economic backgrounds of the population [55]. Furthermore, the type of facility was codified into classes (primary, secondary, and tertiary facilities) according to their manpower, medical equipment, and service provision [56]. Moreover, the score of delay two and delay three, which are delays in reaching and obtaining care [52] was computed similarly to the score of delay one. The score of delay two was computed using four items: namely 1) absence of transportation, 2) expensive cost of transportation, 3) no facility within a reasonable distance and 4) poor road condition. Similarly, the score of delay three was also computed using four items; namely,1) long travel time from health facility to health facility, 2) long waiting time before treatment was received, 3) mistake during an assessment, diagnosis, and treatment and 4) shortage of equipment and supplies. Both delays (two and three) were measured using binary variables and the responses were set as ‘Yes’ and ‘No’ options.

#### Case definition

*Case definition for extended perinatal death*. Death of a fetus born after 28 completed weeks of gestation or neonatal deaths through the first 28 completed days after birth [57].

*Operational definition*. Cases were categorized by the time of death; antepartum, intrapartum, unknown time of stillbirth, and neonatal. Fetal death during the antepartum, intrapartum, and unknown time was taken as the cause of stillbirth in the study. Furthermore, the contributing maternal conditions were classified into five major categories (M1 to M4, with M5 representing no identified condition) per the guidance of ICD-PM-10 (Table 1) [58].

**Table 1:** ICD-PM categories with the specific cause of perinatal death and maternal health condition.

Time of death	Category	Description	Example
Antepartum death	A1	Congenital malformations and chromosomal abnormalities	Anencephaly, encephalocele, microcephaly, congenital hydrocephalus, spina bifida, etc.
	A2	Infection	Congenital syphilis, congenital malaria, congenital rubella syndrome, congenital TB, etc.
	A3	Antepartum hypoxia	Intrauterine hypoxia
	A4	Other specified antepartum disorder	Vasa previa, ruptured cord, twin-twin transfusion, Intraventricular (nontraumatic) haemorrhage, Rhesus and ABO isoimmunization, etc.
	A5	Disorders related to fetal growth	Small for gestational age, macrosomia, post-term, etc.
	A6	Antepartum death of unspecified cause	Intrauterine death of unspecified cause
Intrapartum death	I1	Congenital malformations and chromosomal abnormalities	Anencephaly, encephalocele, microcephaly, congenital hydrocephalus, spina bifida, etc.
	I2	Birth trauma	Intracranial laceration and haemorrhage due to birth injury, Fracture of skull due to birth injury, etc.
	I3	Acute intrapartum event	Intrauterine hypoxia
	I4	Infection	Congenital syphilis, congenital malaria, congenital rubella syndrome, congenital TB, etc.
	I5	Other specified intrapartum disorder	Vasa previa, ruptured cord, twin-twin transfusion, Intraventricular (nontraumatic) haemorrhage, Rhesus and ABO isoimmunization, etc.
	I6	Disorders related to fetal growth	Small for gestational age, extreme low birthweight, macrosomia, post-term, etc.
	I7	Intrapartum death of unspecified cause	Fetal death of unspecified cause
Neonatal death	N1	Congenital malformations, and chromosomal abnormalities	Anencephaly, encephalocele, microcephaly, congenital hydrocephalus, spina bifida, etc.
	N2	Disorders related to fetal growth	Small for gestational age, exceptionally large baby, post-term, etc.
	N3	Birth trauma	Cerebral haemorrhage due to birth injury, intraventricular haemorrhage due to birth injury, etc.
	N4	Complications of intrapartum events	Intrauterine hypoxia, birth asphyxia
	N5	Convulsions and disorders of cerebral status	Neonatal cerebral irritability, neonatal cerebral depression, neonatal coma, etc.
	N6	Infection	Tetanus neonatorum, bacterial meningitis, bacterial sepsis, congenital pneumonia, etc.
	N7	Respiratory and cardiovascular disorders	Respiratory distress syndrome, Neonatal aspiration syndromes, neonatal cardiac failure, neonatal cardiac dysrhythmia, neonatal hypertension, etc.
	N8	Other neonatal conditions	Vasa previa, ruptured cord, twin-twin transfusion, Rhesus and ABO isoimmunization, kernicterus, etc.
	N9	Low birthweight and prematurity	Extremely low birth weight, extreme immaturity
	N10	Miscellaneous	Cases where codes from several other sections of ICD-10 should be used
	N11	Neonatal death of unspecified cause	Congenital renal failure, termination of pregnancy, affecting fetus and newborn, withdrawal symptoms from drug
Maternal conditions	M1	Complications of placenta, cord and membranes	Abruptio placentae, prolapsed cord, chorioamnionitis, etc.
	M2	Maternal complications of pregnancy	Premature rupture of membranes, oligo- and polyhydramnios, ectopic pregnancy, multiple pregnancy, etc.
	M3	Other complications of labour and delivery	Breech delivery and extraction, forceps delivery, Caesarean delivery
	M4	Maternal medical and surgical conditions	hypertensive disorders, maternal injury, maternal use of tobacco, alcohol or drugs, etc.
	M5	No maternal conditions	No condition identified

### Data management and statical analysis

The data was exported from Epi -info version 7.2 to Stata version 17 for data cleaning and further analysis. Using the cleaned data, both descriptive (count and percentage) and analytical analysis (multilevel multinomial logistic regression) were carried out and reported.

#### Model building

In the PDSR data, perinates were nested within a province and it is expected that perinates within the same province are more similar to each other than perinates in the remaining part of the country. Due to the clustered nature of the observations, the assumption of standard regression (which is independent observation and the equal variance among provinces) was violated. In consideration of the gap in standard regression, multilevel multinomial logistics regression was employed to estimate both the independent (fixed) effect of the explanatory variables and the provinces-level (random) effect on the dependent variable [59]. Since the model was nested, model adequacy was carried out based on deviance (−2 log-likelihood). Furthermore, to measure the variability of the timing of perinatal death between provinces, Intraclass correlation coefficient (ICC), median odds ratio (MOR) and proportional change in variance (PCV) were also computed.

In light of the hierarchical nature of the data, two-level mixed effect (multilevel) multinomial logistics regression was applied to explore factors associated with the timing of perinatal death. Four consecutive models were fitted to decide on the final model. The first model was a null model (containing only the outcome variable), the second one was model 1 (model fitted using individual-level variables only), the third was model 2 (model fitted using provincial-level variables only) and the fourth model was model 3 (fitted using individual and provincial variables). The fourth model was selected as the best fitted model due to its lowest deviance value.

Both bivariate and multivariate analyses were employed and variables with P- value less than 0.20 were used as a cut-off point to retain variables for the final multivariable analysis. Multicollinearity between explanatory variables was checked using the Variance Inflation Factor (VIF), which indicates that there was no multicollinearity because all variables had VIF < 5 and tolerance greater than 0.1. Finally, the adjusted Relative Risk Ratio (RRR) with a 95% Confidence Interval (CI) was reported using neonatal death as the reference category of the independent variables. Variables with p- values <0.05, in multivariate analysis, were declared as significant predictors of antepartum, intrapartum, and unknown time of stillbirth.

Furthermore, multilevel multigroup analysis was conducted on selected variables after checking the presence of a difference between a model with distinct parameters and a model with all parameters constrained through a likelihood-ratio test [60].

### Ethical approval

We used secondary data obtained from the EPHI with no personal identifier information of the participants. The EPHI Review Board and Public Health Emergency Management Unit approved the research proposal with Ref. No. EPHI 6_5/437. To keep confidentiality, personal identifiers were not used in the study. Since the study used secondary data sources, consent and other ethical measures were not applicable.

## Result

### Selected characteristics of the reported facilities

A total of 3814 perinatal deaths were reviewed during the study period. Neonatal death has contributed to 62.8% of perinatal deaths, followed by intrapartum stillbirth, unknown time of stillbirth, and antepartum stillbirth, where each contributes 17.5%,14.3%and 5.4% of perinatal deaths respectively. Among the reporting facilities, nearly all perinatal deaths (93.3%) reported by tertiary health care providers occurred during the neonatal period. Region-wise, all death reported from Gambella and Harir regions, took place during the neonatal period. Furthermore, 77.2% of the reported perinatal deaths in the year 2018 occurred during the neonatal period (Table 2).

**Table 2:** Selected background characteristics of reporting facilities by the timing of perinatal death in Ethiopia, 2018–2021.

Variables	Antepartum, N = 203	Intrapartum, N = 666	Unknown time of stillbirth, N = 548	Neonatal, N = 2,397	Overall, N = 3,814
**Type of health facility**					
Primary level of care	160 (8.0%)	486 (24.3%)	345 (17.3%)	1,008 (50.4%)	1,999
Secondary level of care	19 (2.2%)	152 (17.3%)	192 (21.9%)	515 (58.7%)	878
Tertiary level of care	24 (2.6%)	28 (3.0%)	11 (1.2%)	874 (93.3%)	937
**Facility ownership**					
Public facility	201 (5.3%)	661 (17.4%)	548 (14.4%)	2,391 (62.9%)	3,801
NGO	0 (0.0%)	0 (0.0%)	0 (0.0%)	2 (100.0%)	2
Private	2 (18.2%)	5 (45.5%)	0 (0.0%)	4 (36.4%)	11
**Source data**					
FBAF	197 (5.4%)	623 (17.1%)	496 (13.6%)	2,323 (63.8%)	3,639
VA	6 (3.4%)	43 (24.6%)	52 (29.7%)	74 (42.3%)	175
**Reporting region**					
Addis Ababa	10 (1.2%)	25 (3.1%)	12 (1.5%)	761 (94.2%)	808
Amhara	84 (4.2%)	459 (23.1%)	405 (20.4%)	1,041 (52.3%)	1,989
Benishangul Gumuz	8 (11.1%)	21 (29.2%)	3 (4.2%)	40 (55.6%)	72
Dire Dawa	0 (0.0%)	8 (21.1%)	0 (0.0%)	30 (78.9%)	38
Gambella	0 (0.0%)	0 (0.0%)	0 (0.0%)	4 (100.0%)	4
Harir	0 (0.0%)	0 (0.0%)	0 (0.0%)	21 (100.0%)	21
Oromia	78 (13.7%)	127 (22.4%)	116 (20.4%)	247 (43.5%)	568
Sidama	0 (0.0%)	4 (4.2%)	8 (8.3%)	84 (87.5%)	96
SNNPR	23 (14.9%)	16 (10.4%)	1 (0.6%)	114 (74.0%)	154
Somali	0 (0.0%)	6 (9.4%)	3 (4.7%)	55 (85.9%)	64
**Year of reporting**					
2018	35 (7.8%)	38 (8.5%)	29 (6.5%)	346 (77.2%)	448
2019	54 (6.9%)	239 (30.6%)	37 (4.7%)	452 (57.8%)	782
2020	19 (2.2%)	172 (19.6%)	183 (20.8%)	505 (57.5%)	879
2021	95 (5.6%)	217 (12.7%)	299 (17.5%)	1,094 (64.2%)	1,705

### Sociodemographic characteristics of the deceased women

The average maternal parity among women whose perinates died during an unknown time of stillbirth (2.7(SD of 1.79)) was higher as compared to women whose perinates died during the neonatal period (2.3(SD of 1.63)). Besides, the average maternal age among those mothers whose perinate died during the neonatal period (27.7(SD of 5.16)) was higher as compared to women whose perinates died during the antepartum period (26.1(SD of 5.81)). The proportion of intrapartum perinatal death was higher among women who had no education (22.3%) than women who attended a primary level of education (9.5%). The proportion of neonatal death was higher among women who resided in urban areas (74.5%) as compared to women who resided in rural areas (53.3%). Women with complications of the placenta, cord, and membrane had a higher proportion of unknown time stillbirth (29.0%) than women with medical and surgical complications (10.5%) (Table 3).

**Table 3:** Selected background characteristics of the deceased perinate’s mother by the timing of perinatal death in Ethiopia, 2018–2021.

Characteristic	Antepartum = 203	Intrapartum, N = 666	Unknown time still birth, N = 548	Neonatal, N = 2,397	Overall, N = 3,814
**Maternal parity^1^**	2.5(2.02)	2.6(1.92)	2.7(1.79)	2.3(1.63)	2.4(1.73)
**Maternal age^1^**	26.1(5.81)	27.6(5.66)	27.7(5.67)	27.3(5.16)	27.3(5.37)
**Maternal educational status**					
Illiterate	116 (5.4%)	483 (22.3%)	416 (19.2%)	1,153 (53.2%)	2,168
Primary	55 (5.8%)	89 (9.5%)	77 (8.2%)	720 (76.5%)	941
Secondary and above	32 (4.5%)	94 (13.3%)	55 (7.8%)	524 (74.3%)	705
**Religion of the mother**					
Christian	128 (4.3%)	568 (19.1%)	453 (15.2%)	1,829 (61.4%)	2,978
Muslim	73 (9.0%)	96 (11.8%)	93 (11.5%)	550 (67.7%)	812
Traditional	2 (8.3%)	2 (8.3%)	2 (8.3%)	18 (75.0%)	24
**Residence**					
Rural	133(6.3%)	460(21.9%)	387(18.5%)	1118(53.3%)	2,098
Urban	70(4.1%)	206(12.0%)	161(9.4%)	1279(74.5%)	1716
**Maternal health condition**					
Other complications of labour and delivery	5 (3.6%)	48 (34.3%)	20 (14.3%)	67 (47.9%)	140
Maternal medical and surgical conditions	14 (4.7%)	51 (17.3%)	31 (10.5%)	199 (67.5%)	295
Maternal complications of pregnancy	15 (4.6%)	150 (46.0%)	52 (16.0%)	109 (33.4%)	326
Complications of placenta, cord and membranes	27 (6.9%)	86 (22.1%)	113 (29.0%)	163 (41.9%)	389
No maternal conditions identified	142 (5.3%)	331 (12.4%)	332 (12.5%)	1,859 (69.8%)	2,664
**Maternity outcome**					
Alive	184 (5.5%)	474 (14.1%)	454 (13.5%)	2,241 (66.8%)	3,353
Died	19 (4.1%)	192 (41.6%)	94 (20.4%)	156 (33.8%)	461

^1^Mean (SD); n (%)

### Selected characteristics of deceased perinate

The average estimated gestational age was higher among perinate who died during the intrapartum period (36.2(SD of 2.85)) than perinate who died during the neonatal period (35.5(SD of 3.61)). The proportion of perinatal death during the antepartum period was higher among females (6.4%) than males (4.5%). The proportion of perinatal death during the intrapartum period was higher among perinates delivered in transit (42.3%) than perinates delivered in a health facility (16.7%). The proportion of perinatal death was higher among perinates delivered through cesarean section (79.6%) than perinates delivered through spontaneous vaginal delivery (60.1%) (Table 4).

**Table 4:** Selected background characteristics of the deceased perinate by the timing of perinatal death in Ethiopia, 2018–2021.

Characteristic	Antepartum, N = 203	Intrapartum, N = 666	Unknown time of still birth, N = 548	Neonatal, N = 2,397	Overall, N = 3,814
**Estimated gestational age^1^**	35.7(3.01)	36.2(2.85)	35.9(3.17)	35.5(3.61)	35.5(3.42)
**Sex**					
Male	100(4.5%)	384(17.5%)	298(13.5%)	1418(64.5%)	2200
Female	103(6.4%)	282(17.5%)	250(15.5%)	979(60.7%)	1614
**Place of birth**					
Health facility	178(5.1%)	582(16.7%)	460(13.2%)	2258(64.9%)	3478
Home	24(9.1%)	54(20.4%)	68(25.7%)	119(44.9%)	265
On Transit	1(1.4%)	30(42.3)	20(28.2%)	20(28.2%)	71
**Place of death**					
Health facility	109(3.3%)	526(15.7%)	420(12.5%)	2293(68.5%)	3,348
Home	86(24.3%)	88(24.9%)	105(29.7%)	75(21.2%)	354
On transit	8(7.1%)	52(46.4%)	23(20.5%)	29(25.9%)	112
**Mode of delivery**					
Cesarean section	14(2.7%)	58(11.2%)	34(6.5%)	414(79.6%)	520
Operative vaginal delivery	5(2.2%)	51(22.9%)	28(12.6%)	139(62.3%)	223
Spontaneous vaginal delivery	184(6.0%)	557(18.1%)	486(15.8%)	1844(60.1%)	3,071

^1^Mean (SD); n (%)

### Assigned cause of death

The highest proportion (100.0%) of perinatal death was observed during the neonatal period due to complications of intrapartum events, respiratory and cardiovascular disorder, convulsion and disorder of the cerebral status, low birth weight, prematurity, and other neonatal conditions. Similarly, the highest proportion (100.0%) of perinatal death was observed during the antepartum period due to antepartum death of unspecified cause, other specified antepartum disorders, and acute antepartum events (Table 5).

**Table 5:** Assigned cause of death by the timing of perinatal death in Ethiopia,2018–2021.

Characteristic	Antepartum, N = 203	Intrapartum, N = 666	Unknown time of still birth, N = 548	Neonatal, N = 2,397	Overall, N = 3,814
**Assigned cause of death**					
Antepartum death of unspecified cause	1 (100.0%)	0 (0.0%)	0 (0.0%)	0 (0.0%)	1
Intrapartum death of unspecified cause	0 (0.0%)	0 (0.0%)	2 (100.0%)	0 (0.0%)	2
Other specified antepartum disorder	2 (100.0%)	0 (0.0%)	0 (0.0%)	0 (0.0%)	2
Convulsions and disorders of cerebral status	0 (0.0%)	0 (0.0%)	0 (0.0%)	4 (100.0%)	4
Birth trauma	0 (0.0%)	7 (38.9%)	1 (5.6%)	10 (55.6%)	18
Acute antepartum event	25 (100.0%)	0 (0.0%)	0 (0.0%)	0 (0.0%)	25
Other neonatal conditions	0 (0.0%)	0 (0.0%)	0 (0.0%)	25 (100.0%)	25
Respiratory and cardiovascular disorders	0 (0.0%)	0 (0.0%)	0 (0.0%)	110 (100.0%)	110
Congenital malformations, deformations and chromosomal	30 (11.2%)	76 (28.5%)	31 (11.6%)	130 (48.7%)	267
Disorders related to fetal growth	43 (15.7%)	94 (34.3%)	137 (50.0%)	0 (0.0%)	274
Acute intrapartum event	0 (0.0%)	215 (63.4%)	124 (36.6%)	0 (0.0%)	339
Complications of intrapartum events	0 (0.0%)	0 (0.0%)	0 (0.0%)	682 (100.0%)	682
Low birth weight and prematurity	0 (0.0%)	0 (0.0%)	0 (0.0%)	946 (100.0%)	946
Infection	101 (9.0%)	274 (24.5%)	252 (22.5%)	492 (44.0%)	1,119

### Delay factor by the timing of perinatal death

The proportion of neonatal death due to delay one was higher among perinates whose mothers delayed seeking care due to previous bad experiences in health facilities (81.8%) than perinates whose mothers were delayed in seeking care due to lack of decision to a health Facility (42.8%). Similarly, the proportion of unknown time of stillbirth because of delay two was higher among perinates whose mothers were delayed in reaching a health care facility due to poor road conditions (25.2%) as compared to perinates whose mothers were delayed to reach care due to expensive cost of transportation (9.4%). Moreover, the proportion of intrapartum stillbirth because of delay three was higher among women who waited for a longer duration before assessment in a health facility (20.7%) than women who received the wrong diagnosis and treatment (8.0%) (Table 6).

**Table 6:** Delay factors contribute to perinatal death timing in Ethiopia 2018–2021.

Characteristic	Antepartum, N = 203	Intrapartum, N = 666	Unknown time of still birth, N = 548	Neonatal, N = 2,397	Overall, N = 3,814
**Delay 1 –Decision to seek care**					
Family poverty	10 (3.9%)	42 (16.3%)	22 (8.6%)	183 (71.2%)	257
Bad experience with previous health care	0 (0.0%)	1 (9.1%)	1 (9.1%)	9 (81.8%)	11
Failed to recognize the danger pregnancy	116 (10.5%)	222 (20.1%)	163 (14.8%)	603 (54.6%)	1,104
Unaware where to go	6 (11.1%)	9 (16.7%)	11 (20.4%)	28 (51.9%)	54
Had no one take care of other children	2 (4.0%)	9 (18.0%)	9 (18.0%)	30 (60.0%)	50
Reliant on traditional practice	0 (0.0%)	10 (27.0%)	4 (10.8%)	23 (62.2%)	37
Lack of decision to go to health facility	40 (8.8%)	121 (26.7%)	85 (18.7%)	208 (45.8%)	454
**Delay 2 –Reaching care**					
Absence of transportation	7 (3.1%)	53 (23.3%)	37 (16.3%)	130 (57.3%)	227
Expensive cost of transportation	5 (9.4%)	23 (43.4%)	5 (9.4%)	20 (37.7%)	53
No facility within a reasonable distance	4 (3.2%)	36 (28.6%)	13 (10.3%)	73 (57.9%)	126
Poor road condition	2 (1.9%)	24 (23.3%)	26 (25.2%)	51 (49.5%)	103
**Delay 3 –Receiving care**					
Long travel time from health facility to health facility	23 (3.0%)	138 (18.2%)	63 (8.3%)	536 (70.5%)	760
Long waiting time before treatment was received	9 (2.8%)	66 (20.7%)	16 (5.0%)	228 (71.5%)	319
Mistaken during assessment, diagnosis, and treatment	3 (3.4%)	7 (8.0%)	3 (3.4%)	74 (85.1%)	87
Shortage of equipment and supplies	1 (0.5%)	37 (16.8%)	4 (1.8%)	178 (80.9%)	220

### Factors associated with an antepartum time of death among reviewed perinatal deaths

As maternal age increases by one year, the risk of having antepartum stillbirth decreases by 6% [RRR = 0.94;95%CI:(0.90–0.98)]. Perinates who were infected during pregnancy were 5 times more likely to die during the antepartum period as compared to perinates who were not infected [RRR = 5.21;95%CI:(3.58–7.57)]. Perinate who had congenital malformations, deformations, and chromosomal abnormalities were 9 times more likely to die during the antepartum period as compared to perinate who had no congenital malformations, deformations, and chromosomal abnormalities [RRR = 8.71;95%CI:(4.60–14.53)].

As the score of delay one [RRR = 3.69;95%CI:(2.60–5.25)] and delay three [RRR = 5.80;95%CI:(4.28–7.85)] increases by one unit the risk of having antepartum stillbirth increases by 4 and 6 folds for the respective delay factors, while a one-unit increase in the score of delay two decreases the risk of having antepartum stillbirth by 63.0% [RRR = 0.37;95CI:(0.20–0.67)]. The risk of having antepartum stillbirth is 84% [RRR = 0.16;95%CI:(0.07–0.39)] lower among women who were managed in secondary level care than women managed in primary health care. Furthermore, the risk of having antepartum stillbirth is 81% [RRR = 0.19;95%CI:(0.05–0.72)] lower among women who resided in city administration than women who resided in agrarian regions (Table 7).

**Table 7:** Multilevel Multinomial analysis of individual and provincial factors associated with the timing of death among reviewed perinatal death in Ethiopia,2018–2021.

Name of model	Model 2^b^			Model 3^c^			Model 4^d^		
Variables/Characteristics	Individual characteristics			Community characteristics			Individual and Community characteristics		
	Antepartum	Intrapartum	Stillbirth of unknown time	Antepartum	Intrapartum	Stillbirth of unknown time	Antepartum	Intrapartum	Stillbirth of unknown time
	RRR (95%CI)	RRR (95%CI)	RRR (95%CI)	RRR (95%CI)	RRR (95%CI)	RRR (95%CI)	RRR (95%CI)	RRR (95%CI)	RRR (95%CI)
**Maternal age**	0.94(0.90–0.97)**	0.99(0.97–1.02)	0.99(0.96–1.01)				0.94(0.90–0.98) **	0.99(0.97–1.02)	0.99(0.96–1.02)
**maternal parity**	1.13(1.01–1.26)*	1.04(0.97–1.12)	1.06(0.98–1.15)				1.11(1.00–1.25)	1.05(0.97–1.13)	1.06(0.98–1.15)
**Estimated gestational week**	0.98(0.93–1.03)	1.05(1.01–1.08)*	1.02(0.98–1.06)				0.95(0.90–1.00)	1.03(0.99–1.07)	1.01(0.97–1.05)
**Number of ANC visit**	0.98(0.87–1.10)	1.03(0.95–1.12)	0.86(0.79–0.94)***				1.01(0.89–1.14)	1.05(0.96–1.14)	0.86(0.79–0.94)**
**Sex**									
Male ®	*1*	*1*	*1*				*1*	*1*	*1*
Female	1.40(1.02–1.93)*	1.08(0.87–1.34)	1.17(0.94–1.46)				1.29(0.92–1.80)	1.08(0.87–1.34)	1.14(0.91–1.44)
Maternal status									
Alive®	*1*	*1*	*1*				*1*	*1*	*1*
Died	0.75(0.36–1.55)	1.76(1.15–2.68)**	1.18(0.75–1.85)				0.82(0.39–1.72)	1.70(1.11–2.61)*	1.21(0.77–1.92)
**Maternal educational status**									
Illiterate ®	*1*	*1*	*1*				*1*	*1*	*1*
Primary	0.93(0.62–1.40)	0.50(0.36–0.69)**	0.48(0.35–0.67)**				0.73(0.48–1.13)	0.48(0.35–0.66)***	0.52(0.37–0.73)***
Secondary and above	1.17(0.72–1.88)	0.85(0.62–1.18)	0.69(0.48–0.99)*				0.92(0.56–1.51)	0.75(0.54–1.04)	0.70(0.48–1.02)
**Mode of delivery**									
Spontaneous vaginal delivery ®	*1*	*1*	*1*				*1*	*1*	*1*
Operative vaginal delivery	0.55(0.21–1.45)	1.60(1.04–2.46)*	1.10(0.67–1.81)				0.41(0.15–1.09)	1.33(0.86–2.07)	0.95(0.57–1.59)
Cesarean section	0.72(0.39–1.34)	0.84(0.57–1.22)	0.61(0.39–0.94)*				0.70(0.37–1.31)	0.85(0.58–1.24)	0.68(0.43–1.06)
**Birth of place**									
Health facility ®	*1*	*1*	*1*				*1*	*1*	*1*
Home	0.80(0.46–1.40)	1.48(0.97–2.24)	0.96(0.62–1.49)				1.20(0.68–2.13)	1.85(1.22–2.88)**	1.10(0.70–1.71)
On transit	0.36(0.05–2.88)	3.42(1.55–7.52)**	4.53(2.14–9.59)***				0.48(0.06–3.85)	3.58(1.62–7.94)**	4.25(2.02–8.93)***
**Maternal health condition**									
No maternal health condition identified ®	*1*	*1*	*1*				*1*	*1*	*1*
Other complications of labour and delivery	0.61(0.22–1.65)	2.01(1.21–3.31)**	1.05(0.58–1.92)				0.64(0.23–1.79)	1.79(1.07–2.99)*	0.92(0.50–1.71)
Maternal medical and surgical condition	0.84(0.44–1.61)	1.33(0.87–2.03)	0.83(0.51–1.35)				0.79(0.40–1.56)	1.41(0.91–2.18)	0.83(0.50–1.38)
Maternal complications of pregnancy	1.52(0.66–3.47)	2.92(1.81–4.71) ***	1.30(0.75–2.24)				1.73(0.75–4.03)	3.26(2.00–5.29)***	1.31(0.76–2.28)
Complications of placenta, cord, and membranes	1.23(0.72–2.09)	1.13(0.77–1.65)	1.71(1.18–2.48)**				1.26(0.74–2.17)	1.02(0.70–1.51)	1.56(1.08–2.26)*
**Infection**									
No ®	*1*	*1*	*1*				*1*	*1*	*1*
Yes	4.99(3.49–7.14) ***	2.69(2.12–3.41) ***	3.03(2.38–3.87) ***				5.21(3.58–7.57)***	2.73(2.14–3.48)***	2.94(2.29–3.77)***
**Congenital malformations, deformations, and chromosomal abnormalities**							*1*	*1*	*1*
No ®	*1*	*1*	*1*				*1*	*1*	*1*
Yes	6.49(3.75–11.24) ***	4.38(2.89–6.65)***	1.87(1.13–3.10)*				8.17(4.60–14.53)***	4.94(3.22–7.56)***	2.28(1.36–3.82)**
**The score of delay one**	3.33(2.41–4.59) ***	1.53(1.21–1.93) ***	1.16(0.91–1.49)				3.69(2.60–5.25) ***	1.44(1.13–1.84)**	1.40(1.07–1.82)*
**The score of delay two**				0.41(0.23–0.71) **	1.21(0.89–1.66)	1.00(0.71–1.42)	0.37(0.20–0.67)**	1.14(0.81–1.60)	0.95(0.65–1.38)
**The score of delay three**				4.59(3.38–6.07) ***	2.12(1.72–2.61)***	2.41(1.94–2.99) ***	5.80(4.28–7.85)**	2.46(1.96–3.09)***	2.84(2.24–3.60)***
**Level of care**									
Primary health care®				*1*	*1*	*1*	*1*	*1*	*1*
Secondary health care				0.09(0.04–0.21)***	0.21(0.10–0.45)***	0.40(0.18–0.86) *	0.16(0.07–0.39)***	0.24(0.11–0.51)***	0.52(0.25–1.11)
Tertiary health care				0.38(0.12–1.20)	0.08(0.03–0.22)***	0.06(0.02–0.20) ***	0.46(0.14–1.52)	0.08(0.03–0.24)***	0.08(0.02–0.26)***
**Type of region**									
Agrarian ®				*1*	*1*	*1*	*1*	*1*	*1*
Pastoralist				0.93(0.26–3.28)	0.76(0.25–2.31)	0.16(0.04–0.60) **	0.93(0.26–3.28)	0.89(0.30–2.60)	0.17(0.05–0.64)**
City administration				0.20(0.05–0.73)*	0.72(0.26–2.00)	0.36(0.11–1.12)	0.19(0.05–0.72) *	0.94(0.34–2.59)	0.46(0.15–1.44)

*P < 0.05

**P < 0.001

***P < 0.0001

(a) Reference for the dependent variable (neonatal death was the reference for the dependent variable in the study)

® Reference for the category of an independent variable

### Factors associated with an intrapartum time of death among reviewed perinatal deaths

The risk of having intrapartum stillbirth is 52% [RRR = 0.48;95%CI:(0.35–0.66)] lower among women who attended primary level of education than women who had no education. The risk of intrapartum stillbirth is higher among women who give birth at home [RRR = 1.85;95%:(1.22–2.88)] and in transit [RRR = 3.58;95%:(1.62–7.94)] than women who delivered at a health facility. Women with a maternal complication of pregnancy [RRR = 3.26;95CI:(2.00–5.29)] and complications of labour and delivery [RRR = 1.79;95CI:(1.07–2.99)] had a higher risk of having intrapartum stillbirth as compared to women with no identified risk factors. Perinate who were infected during pregnancy were 3 times more likely to die during the intrapartum period as compared to perinate who were not infected [RRR = 2.73;95%CI:(2.14–3.48)]. Perinate who had congenital malformations, deformations, and chromosomal abnormalities were 5 times more likely to die during the intrapartum period as compared to perinate who had no congenital malformations, deformations, and chromosomal abnormalities [RRR = 4.94;95%CI:(3.32–7.56)]. As the score of delay one [RRR = 3.69;95%CI:(2.60–5.25)] and delay three [RRR = 2.46;95%CI:(1.93–3,09)] increases by one unit the risk of having intrapartum stillbirth increases by four and three folds with the respective categories. In addition, the risk of having intrapartum stillbirth is 76% [RRR = 0.24;95%CI:(0.11–0.51)] lower among women who were managed in secondary level care than women who were managed in primary health care. Similarly, the risk of having intrapartum stillbirth is 92% [RRR = 0.08;95%CI:(0.03–0.24)] lower among women who were managed in a tertiary level health care than women who were managed in the primary level of care (Table 7).

### Factors associated with an unknown time of death among reviewed perinatal deaths

As the number of ANC visits increases by one unit the risk of having an unknown time of stillbirth is decreased by 14% [RRR = 0.86;95%CI:(0.79–0.94)]. The risk of having an unknown time of stillbirth is 48% [RRR = 0.52;95%CI:(0.37–0.73)] lower among women who attended primary level of education than women who had no education. As compared to women who give birth in a health facility, the risk of having unknown time stillbirth is higher among women who deliver while in transit [RRR = 4.25;95%CI:(2.02–8.93)]. The risk of having unknown time stillbirth is 48% [RRR = 1.56;95%CI:(1.08–2.26)] higher among women with a complication of the placenta, cord, and membranes than in women with no identified complication. Perinates who were infected during pregnancy are three times more likely to die during an unknown time of stillbirth as compared to perinates who were not infected [RRR = 2.94;95%CI:(2.29–3.77)]. Perinates who had congenital malformations, deformations, and chromosomal abnormalities were two times more likely to die during an unknown time of stillbirth as compared to those who had no congenital malformations, deformations, and chromosomal abnormalities [RRR = 1.40;95%CI:(1.07–1.82)]. As the score of delay one [RRR = 3.69;95%CI:(2.60–5.25)] and delay three [RRR = 2.84;95%CI:(2.24–3.60)] increases by one unit the risk of having intrapartum stillbirth increase by four and three folds with respective categories. Furthermore, the risk of having an unknown time of stillbirth is 92% [RRR = 0.08;95%CI:(0.02–0.26)] lower among women who were managed at the tertiary health care level than women managed at the primary health care level. Lastly, women who resided in the pastoralist area had an 83% [RRR = 0.17;95%CI:(0.05–0.64)] lower risk of having an unknown time of stillbirth than women who resided in the agrarian region (Table 7).

### Multilevel analysis (random-effects analysis)

The status of the time of death varied across provinces (τ^2^ = 3.20, p = <0.001). The empty model revealed that 49.1% of the total variance in time of death was accounted for by between-cluster variation of characteristics (ICC = 0.491). The provinces’ variability declined over successive models, from 49.1% in the empty model to 46.6% in the individual-level-only model, 39.6% in the provinces-level-only model, and 36.5% in the final (combined) model. PCV for the final model was 40.4%, which indicates that 40.4% of the variability in the timing of perinatal death is explained by both individual and provincial-level factors. In addition, the median odds ratio (MOR) in all models was greater than one, demonstrating that there is a variation in the timing of perinatal death between provinces. The MOR was 5.43 in the null model, which indicates the presence of high variation between provinces in the timing of perinatal death (Table 8).

**Table 8:** Results from the random intercept model (a measure of variation) for the timing of death at the province level using multilevel logistic regression analysis.

Random effect	Model_1^a^	Model_2^b^	Model _3^c^	Model_ 4^d^
Provinces level variance (SE)	3.17(0.5)	2.64(0.5)	2.18(0.4)	1.89(0.4)
P_values	<0.001	<0.001	<0.001	<0.001
ICC (%)	49.1%	44.60%	39.8%	36.5%
**MOR**	5.43	4.69	4.06	3.70
Explained variance (PVC) (%)	Reference	16.70%	31.2%	40.4%
**Model fit statics**				
log like hood	-3301.57	-3029.47	3147.31	-2883.28
AIC	6611.14	6180.94	6338.62	5924.56
BIC	6636.13	6561.97	6476.04	6418.03

#### Model fit statistics

As shown in Table 8 (model fit statistics), the values of the Akaike information criterion (AIC) and Bayesian Information Criterion (BIC) showed subsequent reduction which indicates that each model represents a significant improvement over the previous model, and it points to the goodness of fit of the final model built in the analysis.

### Multigroup analysis

Multigroup analysis was conducted using the type of region on the selected variable, which was found to have different values after checking the likelihood-ratio test. The risk of perinatal infection resulting in antepartum stillbirth is higher among perinates whose mothers resided in city administration [RRR = 6.62;95%CI:(1.84–23.78)]. Besides, the risk of perinatal infections causing intrapartum stillbirth is higher among perinate whose mothers resided in the agrarian region [RRR = 3.18;95%CI:(2.26–4.48)]. Lastly, the risk of perinatal infection resulting in an unknown time of stillbirth is higher among perinate whose mothers resided in pastoralist regions [RRR = 14.90;95%CI:(2.16–42.62)]. Furthermore, the risk of perinatal congenital malformations, deformations, and chromosomal abnormalities resulting in antepartum stillbirth is higher among perinate whose mothers resided in the agrarian region [RRR = 9.02;95%CI:(3.01–28.11)]. Furthermore, the risk of perinatal congenital malformations, deformations, and chromosomal abnormalities causing intrapartum stillbirth is higher among perinate whose mothers resided in the pastoralist region [RRR = 5.55;95%CI:(1.26–24.46)] (Table 9).

**Table 9:** Multi-group analysis by type of region within selected variables related to perinatal death in Ethiopia,2018–2021.

Selected variables				
** Timing of death**	Neonatal^(a)^	Antepartum	Intrapartum	Stillbirth of unknown time
** **		RRR (95%CI)	RRR (95%CI)	RRR (95%CI)
**Pastoralist region(n = 140)**				
Infection				
Yes		5.11(1.65–15.84) *	3.07(1.24–7.55) *	14.90(2.16–42.62) *
No®		*1*	*1*	*1*
Congenital malformations, deformations, and chromosomal abnormalities				
Yes		0.15(0.04–0.24) ***	5.55(1.26–24.46) *	9.96(0.42–238.13)
No ®		*1*	*1*	*1*
**City administration(n = 867)**				
Infection				
Yes		6.62(1.84–23.78) **	1.27(0.10–16.20)	5.70(1.55–21.03) **
No ®		*1*	*1*	*1*
Congenital malformations, deformations, and chromosomal abnormalities				
Yes		0.26(0.04–0.60) **	3.47(0.05–247.22)	6.74(0.82–55.65)
No ®		*1*	*1*	*1*
**Agrarian (n = 2807)**				
Infection		5.74(2.27–14.52) ***	3.18(2.26–4.48) ***	4.00(2.25–7.12) ***
Yes		*1*	*1*	*1*
No ®				
Congenital malformations, deformations, and chromosomal abnormalities				
Yes		9.20(3.01–28.11) ***	3.88(2.15–7.01) ***	2.79(1.49–5.22) **
No ®		*1*	*1*	*1*

*P < 0.05

**P < 0.001

***P < 0.0001

® Reference for the category of an independent variable

^(a)^ Reference for the dependent variable

## Discussion

In general, the study revealed that both individual (perinatal and neonatal) and provincial factors had an important role in determining the timing of perinatal death. Maternal age, number of ANC visits, maternal education, place of birth, maternal health condition, cause of death (infection and congenital and chromosomal abnormalities) and delay one were included under individual-level factors. While delay two, delay three, type of health facility, and type of region were included under provincial-level factors associated with the timing of perinatal death.

The study revealed that more than half of the perinatal deaths occurred during the neonatal period followed by the intrapartum period and unknown time of stillbirth. The timing of death for a significant number of stillbirths was not determined and was stated as the “unknown time of stillbirth”. This indicates the status of the country’s diagnostic capacity (autopsy, histological examination of the placenta, swabs, cultures, and minimal maternal blood tests) and stillbirth auditing performance [61]. In addition, assigning the timing of perinatal death solely based on physical appearance (i.e., fresh, and macerated stillbirth), is not an accurate parameter to determine the timing of perinatal death, rather it can be used as a proxy indicator. Henceforth, the classification of perinatal mortality based on the timing of death should be supported by fetal heart rate findings along with physical appearance to properly classify and eventually reduce preventable perinatal death [62].

The study revealed that maternal age is an important factor in perinatal death. According to the study findings, increased maternal age is associated with a decreased risk of having an antepartum stillbirth. The finding was parallel with studies conducted in Ethiopia [63] and Tanzania [34]. This might have a connection with maternal age at first birth, which is below 20 years in the Ethiopian context [64]. Thus, as maternal age at first birth increases the chance of school dropout is reduced and this paves the way to obtain vocational skills that improve the economic status of the mother, her child, and the family as well [65]. Nevertheless, there is ample evidence suggesting that women who are 35 years and above will have adverse maternal and fetal outcomes [41, 66].

The study also revealed that as the number of ANC visits increases the risk of having an unknown time of stillbirth decreases. The finding was concurrent with previous studies conducted in Ethiopia [67, 68], Kenya [69], and Nepal [40]. The plausible explanation might be related to the frequent ANC visits’ ability in identifying potential risk factors that contribute to stillbirth [70]. However, in Ethiopia, only 43% of women had more than four ANC visits during their last pregnancy [50].

Maternal education is the other variable that has an integral role in perinatal death. Women with primary education had a lower risk of having intrapartum and unknown time of stillbirth than women with no education. Besides, a positive dose response was observed between the timing of perinatal death and the score of delay one. The finding was congruent with studies conducted in Ethiopia (Baharidar and Bale) [68, 71], Bangladesh [72], Jordan [45], Nepal [73], and India [74]. This might be due to illiteracy’s role in compromising the economic status, access to health care, and birth spacing of the mother. The combination of all these factors will lead to deteriorated maternal and fetal outcomes. Although 40% of pregnant women in Ethiopia are not educated [50], the country has made a remarkable stride on this front by establishing a health extension program, to avail health education and basic health service at the community level [75]. Overall, the findings implied that health education should still be considered as a key pillar when prioritizing interventions that are aimed at improving perinatal outcomes.

Compared to home and in transit, delivery in a health facility reduces the risk of having an intrapartum and unknown time of stillbirth. The finding was coherent with studies conducted in five countries (India, Guatemala, Kenya, Pakistan, and Zambia) [76]. This is mainly due to the ability of health facilities to provide service under the watch of a health care professional, who can manage normal labour and delivery and identify complications or provide basic care and referral. In addition, women who give birth in a health facility, with basic emergency obstetric and newborn care (BEmONC), had the potential to avert intrapartum stillbirth by 45%; the probability is further reduced by 75% when women attend delivery within a health facility providing comprehensive basic emergency obstetric and newborn care (CEmONC) services [77]. However, the use of health facilities for delivery is still very low in Ethiopia, where only 48% of pregnant women deliver in a health facility [50].

Maternal health conditions are the other factor that had a significant role in determining the intrapartum and unknown time of stillbirth. Maternal complications of pregnancy, complications of labour and delivery as well as complications of the placenta, cord, and membranes were mentioned as the leading cause. The finding was congruent with studies conducted in Ethiopia [78], Uganda [79, 80], Tanzania [81], India [33], Jordan [82], and Taiwan [83]. The plausible explanation for this might be related to uteroplacental hypoperfusion, which results in fetal distress during the intrapartum period leading to fetal death. Lack of ANC visit, urinary tract infection, nutritional status, vaginal bleeding, maternal health condition (uncontrolled gestational diabetics and hypertension), previous cesarean section, and prolonged labour were modifiable risks for the three above mentioned maternal conditions [84–88]. Maternal health conditions could be identified and managed through the provision of quality pre-conception care, ANC care, and intrapartum care. The provision of these services should, among others, include prenatal ultrasound scanning, which has a positive perinatal outcome in a low-resource setting [89]. In Ethiopia, the quality of ANC service is being compromised due to the limited coverage of prenatal Ultrasound and a lack of trained personnel [90, 91]. Overall, the finding demonstrated that the country has a long way to go in improving the quality of service from the period of conception up to delivery.

The study also revealed that Infection has a positive relation with antepartum, intrapartum, and unknown time of stillbirth. The finding was parallel with studies conducted in Ethiopia [92], Ghana [93], South Africa [94], Bangladesh [95], China [96], and Afghanistan [97]. This might be explained by infection’s role in causing stillbirth through different mechanisms, including direct infection, placenta damage, and severe maternal illness. These all deteriorations result in stillbirth by damaging vital organs, obstructing blood flow, and diminishing oxygen supply [98]. In line with this, per a recent study, nearly 32% of stillbirths in sub-Saharan Africa are caused by infection, specifically, malaria, syphilis, and HIV Aids [99]. Similarly, the prevalence of syphilis and malaria was significant among pregnant women in Ethiopia [100, 101]. In addition to those factors, Streptococcus colonization has a significant role in stillbirth outcomes [102]. Therefore, interventions such as syphilis screening and treatment in combination with malaria prophylaxis should be well integrated into the routine ANC service to reduce the burden of stillbirth in the country [103]. Maternal vaccination should also be considered as a mitigation strategy to improve perinatal outcomes [104, 105].

Congenital malformations, deformations, and chromosomal abnormalities were also positively related to the three-timing of perinatal deaths. The finding was coherent with studies conducted in Ethiopia (Bahardar and North Shewa) [71, 106] Cameroon [107], India [33], and Jordan [108]. This could be explained by the organ dysfunction as well as the lack of vital organs used for the survival of the fetus making them more vulnerable to fatal complications. These cascades of complications result in death during the intrauterine life of the fetus. Advanced maternal age, residence, pre-existing maternal health condition, ANC visit (intake of iron folate and multivitamin), exposure to pesticides, maternal smoking status, and taking teratogenic medication during the first three months of pregnancy were identified as risk factors resulting in congenital anomalies in Ethiopia [109–114]. To this effect, WHO recommends the establishment of birth defect surveillance to track, assess and improve the management of congenital anomalies at individual and national levels [115]. However, Ethiopia has not made any progress to establish the surveillance system yet [116].

The type of health facility and delay three were the other variables associated with the timing of stillbirth. The risk of antepartum, intrapartum, and unknown time of stillbirth was higher among women who were treated in primary health care facilities. Besides, positive dose-response relation was observed between all timing of perinatal deaths and the score of delay three. Those findings were comparable with studies conducted in Ethiopia [68], Nigeria [117], Gambia [118], Zanzibar [119], and India [120]. The plausible explanation for this could be related to the limited capacity of lower-level health facilities in early identifying and managing antepartum and intrapartum complications including referral services. This argument is well supported by the Ethiopian Service Availability and Readiness Assessment (SARA), where tertiary and secondary level health facilities have shown better service availability and readiness in terms of Emergency and Essential Obstetric and Newborn care (Em/EON) [121]. In addition, the country has invested a lot in the establishment of neonatal intensive care units (NICU) at selected secondary and tertiary level facilities [122]. On a related note, In Ethiopia, neonatal referral is being challenged by lack of transposition and effective communication, as well as poor adherence to referral protocols [123]. Acknowledging this gap the country has introduced a clinical mentoring and coaching program to boost the quality of health service provision at lower-level facilities under close follow and support of teaching hospitals [124]. Despite all this attempts, however, the overall study finding suggested that the country has an unfinished task in improving the quality of service by availing trained personnel along with essential equipment.

Lastly, inter-regional variation was observed during the antepartum and unknown time of stillbirth. The multi-group analysis reviled that a significant intra-regional variation was observed among regions in the two causes of death (infection and congenital anomalies). The finding corresponded well with previous studies conducted in Ethiopia [125], Afghanistan [97], Nepal [126], Brazil [127], and China [128]. The sub-national variation of stillbirth could be explained due to the inequality in socioeconomic status as well as the geographical distribution of health services [129]. In addition, significant regional variation was observed in terms of Em/EON service availability and readiness [121]. Overall, the finding demonstrated that national and subnational health policies and resources should be aligned to achieve the target put forward at national and global levels in reducing perinatal mortality [130].

The study had several limitations that need to be acknowledged. 1) the study primarily used surveillance data, which had limited coverage and completeness at the community level, and this could introduce potential bias into the study. 2) considering the national estimate of perinatal death, the surveillance data has only captured the tip of the iceberg. 3) lack of national guidelines and capacity for diagnostic assessment of stillbirth, including routine blood tests, culture, radiology, and minimally invasive autopsy influenced the study in obtaining more in-depth data.

## Conclusion

Almost six out of ten perinatal deaths occurred during the neonatal period, followed by intrapartum perinatal and unknown time of stillbirth. The timing of perinatal death was influenced by both individual and facility-level factors. Maternal (maternal age, ANC visit, maternal education, maternal health condition, delay one), neonatal (place of birth and cause of death), and health facility (delay three, type of facility, and type of region) had a significant role in determining the timing of perinatal death in Ethiopia. Efforts are needed on community-based education and mobilization to reduce the burden of stillbirth by enhancing community awareness around ANC service utilization, institutional delivery, and danger signs of pregnancy. In addition, special attention and more targeted strategies are required for the two (infection and congenital anomalies) significant cause of death to reduce the burden of perinatal death in Ethiopia. Besides, a concerted effort by all stakeholders is needed to improve the coverage and content of ANC services for early identification and management of high-risk pregnancies along with early referral with special attention to lower-level facilities and selected regions.
